# One Health of Peripheries: Biopolitics, Social *Determination*, and Field of Praxis

**DOI:** 10.3389/fpubh.2021.617003

**Published:** 2021-06-30

**Authors:** Oswaldo Santos Baquero

**Affiliations:** ^1^Department of Preventive Veterinary Medicine and Animal Health, School of Veterinary Medicine and Animal Science, University of São Paulo, São Paulo, Brazil; ^2^Research Group on Peripheries, Institute of Advanced Studies, University of São Paulo, São Paulo, Brazil

**Keywords:** one health of peripheries, one health, collective health, critical epidemiology, social determinants of health, health inequities, more-than-human biopolitics, critical animal studies

## Abstract

Amid the urgency to solve countless and severe health problems, asking what is health or who can and must have it may seem like a waste of time. However, some responses can reveal prevailing practices that divert attention from fundamental problems, thus maintaining privileges and deepening health inequities. One Health of Peripheries arises from these questions and takes three interdependent senses. The first refers to attributes determining the well-being and suffering of peripheral multispecies collectives: a state, a process, the realization of capacities. The second problematizes marginalizing apparatuses that define health and who can and should have it. The third encompasses practices in more-than-human social spaces in which, and through which, One Health is experienced, understood, and transformed. The qualification of health as “one” does not refer to the lack of plurality, nor to the simple aggregation of health fragments (human + animal + environmental), but to the complexity of health in a field with peripheral places, ensuing from margins to privilege those who are inside and legitimize the exploitation of those who are outside. The interaction among margins creates degrees and kinds of privilege and vulnerability that materialize epidemiologic profiles while articulating different peripheral strengths and needs supports a collective resistance to break margins. Social *determination*, a key concept in the (Latin American) collective health movement, underlies such profiles. However, this movement overlooks the more-than-human dimension of social determination; that is to say, One Health of Peripheries is a blind spot of collective health. The cartography of One Health of Peripheries has unique needs regarding participation, research, and inclusive policies for the decolonial promotion of healthy lifestyles.

## Introduction

What is health, who can be healthy, and what are the most pressing health issues? I will argue that prevailing answers so far have been biased by struggle, cooperation, and imposition to shape and legitimize hierarchies according to the interest of the most privileged hierarchical positions.

Conceptual frameworks about the social *determination* of health (1, 2) and the social determinants of health (3) consider social hierarchies, giving us insights and tools to oppose specific health inequities. However, one of my claims in this paper is that at the same time, these frameworks ignore and reproduce some marginalizing apparatuses that materialize more-than-human health inequities.

Drawing from Foucault (4) and Agamben (5), I take as apparatus the system of relations between discursive practices, institutions, and more generally, anything with the capacity to determine, control, model, or administer living beings. By marginalizing apparatuses, I mean those that establish margins and legitimize the exploitation and violence against living beings at the other side of the margins, attributing to them and their interests less value while silencing their resistance and agency.

Peripheries are beyond the margins. Patriarchy margins create gender peripheries, just as species margins produce species peripheries. The same happens with racial, ethnic, and geographic margins, among others.

The (Latin American) collective health field (6, 7) has been concerned with some peripheries but systematically produces and reproduces apparatuses that marginalize non-human animals (hereafter animals). In collective health, animals have instrumental value to prevent and control specific human health problems. However, they do not figure as health bearers or in any other explicit form in its conceptual frameworks about the social determination of health. Although such marginalization is common to different health perspectives, I will focus my critic on the collective health field because it is one of the main influences on One Health of Peripheries.

Is the marginalization of animals from the field of collective health justified? I will conclude that it is not. The bourdieusian's approach that supports this field (6) and critical analysis of social hierarchies (8) also shows, together with other perspectives, the more-than-human dimension of social entanglements (9–12). Moreover, concerns with health inequities can be better addressed considering theories of multispecies justice (13), while labor perspectives of health [see Almeida-Filho's discussion about Laurell's works (14)] could be updated by more-than-human labor theories (15).

Health is not exclusively human, as demonstrated by the overwhelming One Health scientific evidence about the human-animal-environment interface (16). One Health is supported by intersectoral and international initiatives due to its pertinence to address pandemics, bioterrorism, food-borne diseases, and significant health problems expected to worsen, such as antimicrobial resistance (16, 17). However, One Health approaches often omit social processes from empirical analysis and theoretical explanations. They encourage intersectoral collaboration as if it were a matter of symmetrical negotiation between institutions, or even more problematic, a matter of global North assistance for the global South (18).

The biologism in One Health has remarkable exceptions (19–25). Here I propose another one: One Health of Peripheries. I rethink One Health from the perspective of Latin American collective health, more-than-human biopolitics, and other critical approaches. Inevitably, this brings together contradictions and some incommensurable aspects. However, we must embrace these challenges instead of assuming that we can translate convenient solutions for ideal settings to a real-world full of contradictions and power relationships, far from being a coordinated network of rational actors.

The epistemologies of the South offer us alternatives such as the ecology of knowledge (26) and hybrid cultures (27), among others, to think complexity, contradiction, plural knowledge, and intercultural translation. That said, my objective here is not to make remarkable advancements in epistemic translation. Instead, in this paper, I point to some conceptual tools that help to identify peripheries and break margins. It is a starting point to introduce One Health of Peripheries, its social determination, and an explicit commitment to advance structural alternatives for multispecies justice. In a separate paper, we elaborate more on the ecology of knowledge, the decolonial stance of One Health of Peripheries, and seven actions to promote the health of marginalized multispecies collectives (18).

The following sections of the paper sketch the emerging field of One Health of Peripheries. A field requiring new practices and policies as well as including other actions already existing but applied elsewhere. Notwithstanding the relevance, my objective here is not to address specific procedures to conduct health practices or concrete recommendations to guide health policies. The *more-than-human biopolitics* section locates marginalizing apparatuses in a broader biopolitical field. It then outlines the role of domestication and animalization in the establishment and operation of hierarchies that determine epidemiologic profiles; it also elaborates on the intersection of margins as well as on possibilities of resistance. The One *Health* section rethinks One Health and draws initial cartography of its peripheral regions. The *social determination of health* section briefly compares the concepts of social determination of health and social determinants of health. From this comparison and the previous sections, I extend the idea of triple inequity of health to include other forms of inequities and their interactions, with particular attention to species-based inequities. The *field of praxis* section is based on Bourdieu's concepts of *habitus* and *field* and Freire's understanding of praxis. In that section, I frame One Health of Peripheries as a blind spot of collective health. Finally, I present some concluding remarks.

## More-Than-Human Biopolitics

Biopolitics addresses new forms of power or aspects of power previously unknown, in the context of phenomena as diverse as concentration camps, migratory processes, cognitive capitalism, domestication, sovereignty, the immunitary paradigm of modern politics, the relationship of humans with others animals and with technology, the state of exception, and power/knowledge relationships (4, 28–37). Such diversity brings ambivalence and contradiction as well as negative (marginalizing, excluding, repressing) and positive (affirmative, productive, empowering) perspectives. Biopolitics shows the blurring of the public/private boundary, the politics on life and of life, the administration of populations, the production of profitable and docile bodies, and marginalizing apparatuses underlying hierarchies (31, 36, 38).

Here it is convenient to come back to the notion of apparatus as the system of relations between discursive practices, institutions, and more generally, anything with the capacity to determine, control, model, or administer living beings. This notion is related to the authorities of delimitation (39)—“including philosophical, religious, scientific and legal”—that delimit and authorize margins and legitimize their practices (40). As one can read in Derrida (41), the original marginalization is constitutive of the socialization of “human culture and of politics itself”; it is a marginalization that leaves animals on the periphery and allows their domestication. Such domestication gives rise to disciplinary and violent regimes (40) and to population technologies for the administration of life. It becomes a model of exploitation and establishes the basis of hierarchical orderings.

Animal domestication required demographic technologies to control population densities, a complex mixture of enforcement and behavioral tactics to administer animal resistance, and care procedures to sustain life. The ensuing more-than-human social relationships established a complex network of codetermination. Demographic technologies for animals allowed human demographic processes of growth and specialization. Food surplus stimulated the formation of storage specialists, leading to positive feedback on food surplus and available time for the emergence of population administrators, accumulation experts, and bureaucrats (42). Animals were not the unique target of the mentioned mixture.

The increasing size and complexity of multispecies settlements was the basis for further social differentiation, unequal distribution of resources, and colonization (42, 43); a process resting on the war against animals (44), the domestication of human collectives, and technologies of accumulation.

Domestication also determined another phenomenon of relevance for more-than-human health. Higher multispecies densities set an appropriate scene for emerging zoonoses and epidemics. So domestication is also a history of epidemics, turned into pandemics by colonization.

The biopolitics of domestication is not a finished remote history. Medical textbooks for 19th-century landowners described procedures to reproduce slaves and increase their productive efficiency, in many respects indistinguishable of current livestock production procedures: compartmentalization of facilities; populations divided according to demographic criteria of productive and reproductive interest; classification and monitoring of morbidity and mortality; prevention of communicable diseases; reproductive selection (genetic improvement); hygiene, nutrition, socialization, and other generic practices to reduce losses of biological capital [see the documented analyses of such practices by Smithers and Camacho (45, 46)]. In the 20th century, the anti-Semite Henry Ford talked in his autobiography about the disassembly line of a Chicago slaughterhouse that inspired his assembly-line method (47, 48), which in turn informed assembly lines to kill Jews in Nazi Germany (30, 48). In the current century, big data and artificial intelligence fuel genetic and molecular interventions and disease surveillance across species, sophisticating biopolitics and further blurring binary distinctions: natural/artificial, human/non-human, public/private.

Marginalizing apparatuses come into play when biopolitics inflict suffering and produce privileges. They are constituents of speciesist, racist, ethnic, class, gender, capacity, and geographic marginalization. Furthermore, the interaction among marginalizing apparatuses creates more peripheries.

Animalization is a marginalizing apparatus applied to some human groups. As recently as 1920, the Wildlife Conservation Society (the same institution that decades later proposed the One Health concept) was responsible for exhibiting Ota Benga, a young black man, at the Bronx Zoo (49). Pugliese makes a “deanthropocentric” reading of Foucault's Madness and Civilization to argue that the lack of rationality operated the animalization of the so-called mad people, justifying their confinement and physical restriction (40). Besides these and other conspicuous examples of animalization, more nuanced practices reinforce human marginalization (think in everyday language). Moreover, animalization also operates in animals, establishing a category of exploitable beings for human benefit.

It is worth noting that animalization does not consistently downplay animals. Sometimes “animal” features are exalted and attributed to humans (fondness, strength, agility) while “human” characteristics (criminal, terrorist, beggar) justify violence against certain human groups. Animalization is inherently aporetic as it operates on who is already an animal, whether human or not. Furthermore, animalization is not involved in all cases of human marginalization.

The interaction between marginalizing apparatuses encompasses more than animalization. Social class determines the material resources of multispecies households. The opportunities for humans and animals (especially the fate of farm animals) are conditioned by disability and sex. Gender is strongly associated with animal protection advocacy. Concentrated Animal Feeding Operations exploit animals and hire marginalized ethnic groups to do unhealthy jobs (50). The racial marginalization of human communities affects the multispecies collectives with which those humans entangle.

The above examples show that some margins directly intersect each other only in humans, others intersect in humans and animals, while others simultaneously segregate multispecies collectives. Race, gender, class, and ethnic margins rest on human attributes, and through them, they affect multispecies collectives. Species, sex, and disability margins target human and animal subjects. Geographic margins segregate multispecies collectives.

The examples are gross simplifications of more complex intersections. A Black non-heteronormative woman living in a favela and protecting animals faces the burden of multiple margins that compromise the capacity to care for her animals. Worsened animal health and insufficient reproductive control increase the psychological and economic demands, while zoonotic spread and animal overpopulation exacerbate the community burden. Moreover, many humans residing in favelas were small farmers displaced by agribusiness apparatuses that at the same time have devastating consequences for traditional communities, wildlife, and exploited farm animals and workers.

The idea of intersecting margins is not new. It is at the core of intersectionality, which emerged to address the legal limitations to repair injustices suffered by Black women (51). One of the claims of intersectionality is that the marginalization of black women is not the sum of sexist and racist burdens; sex discrimination is not equally experienced by Black and White women, just as racial discrimination differs between Black men and women (51). Intersectionality has evolved among scholars and activists, bringing together awareness, confusion, overuse, and deeper explanations. The multiplicity of intersectional concerns has grown because there are many heterogeneous marginal experiences.

The overlay of peripheries produces particular experiences of marginalization and resistance without requiring that attributes of direct marginalization are present in the same individual (as some examples above showed). Furthermore, different peripheries share borders, giving rise to a remarkable possibility: articulating each periphery's strengths and needs supports a collective resistance not to turn hierarchies upside-down but to break margins. Thus, marginalized multispecies collectives can strengthen intersectionality and benefit from it, but that requires effective articulation, a non-trivial task.

Earlier, I mentioned accumulation experts and accumulation technologies. Later, the examples of multispecies intersectionality implicitly showed that capitalism is a shared marginalizing apparatus, that is to say, a common target of intersectional resistance. The biopolitics of animal populations was a condition of possibility for human biopolitics, colonization, and capitalism. These, in turn, reinforced and sophisticated animal biopolitics and produced other marginalizing apparatuses. Therefore, what is at stake is far from being a unidirectional process. A complex network of power relationships constantly moves margins in multiple directions, so individual and collective experiences of marginalization are also dynamic.

Marginalizing apparatuses mobilize exploitation, care, administration, discipline, subjectification, resistance, affects, and legitimization. They produce and reproduce peripheries that partially determine the health experience of multispecies collectives.

## One Health

One health traditionally refers to the inextricable relationship between human, animal, and environmental health. It is a concept growing in popularity and application due to the increasing awareness regarding many human diseases with an animal origin and the multiple diseases that remain zoonotic; from AIDS to dengue and COVID-19, from visceral leishmaniasis to tuberculosis and influenza A (52–54). According to the World Organization for Animal Health (OIE), 60% of human infectious diseases are zoonotic, 75% of emerging human infectious diseases originate from other animal species, and 80% of agents with bioterrorist potential are zoonotic (17). Neglected tropical diseases are mostly zoonotic or vector-borne (55) and affect more than a billion people (56) as well as a high number of animals. Neglected tropical diseases are a priority recognized by the World Health Organization (WHO), particularly in its road map for 2021–2030, which recommends One Health approaches, to attain the Sustainable Development Goals (57). In the face of growing global concern about emerging and re-emerging zoonoses and antimicrobial resistance due to indiscriminate overuse of antibiotics in human populations and other species, One Health catalyzed the tripartite union between the WHO, the OIE, and FAO (16). More recently, One Health approaches entered in the general and specific objectives of the European Union *Programme for the Union's action in the field of health (“EU4Health Programme”) for the period 2021–2027* (58).

One Health is often represented as three partially intersected sets (human, animal, environment). Thus, although humans and animals are *within* the environment, part of the human and animal sets is outside it. Furthermore, the partial intersection between the human and animal domains is incongruent with evolutionary theory since humans are animals. Of course, representations can emphasize different issues; however, there is no need to leave part of the sets out of the intersection. Subsumption serves to represent the relationships, and it is in line with the inclusiveness required to promote One Health of Peripheries.

**Figure 1:**
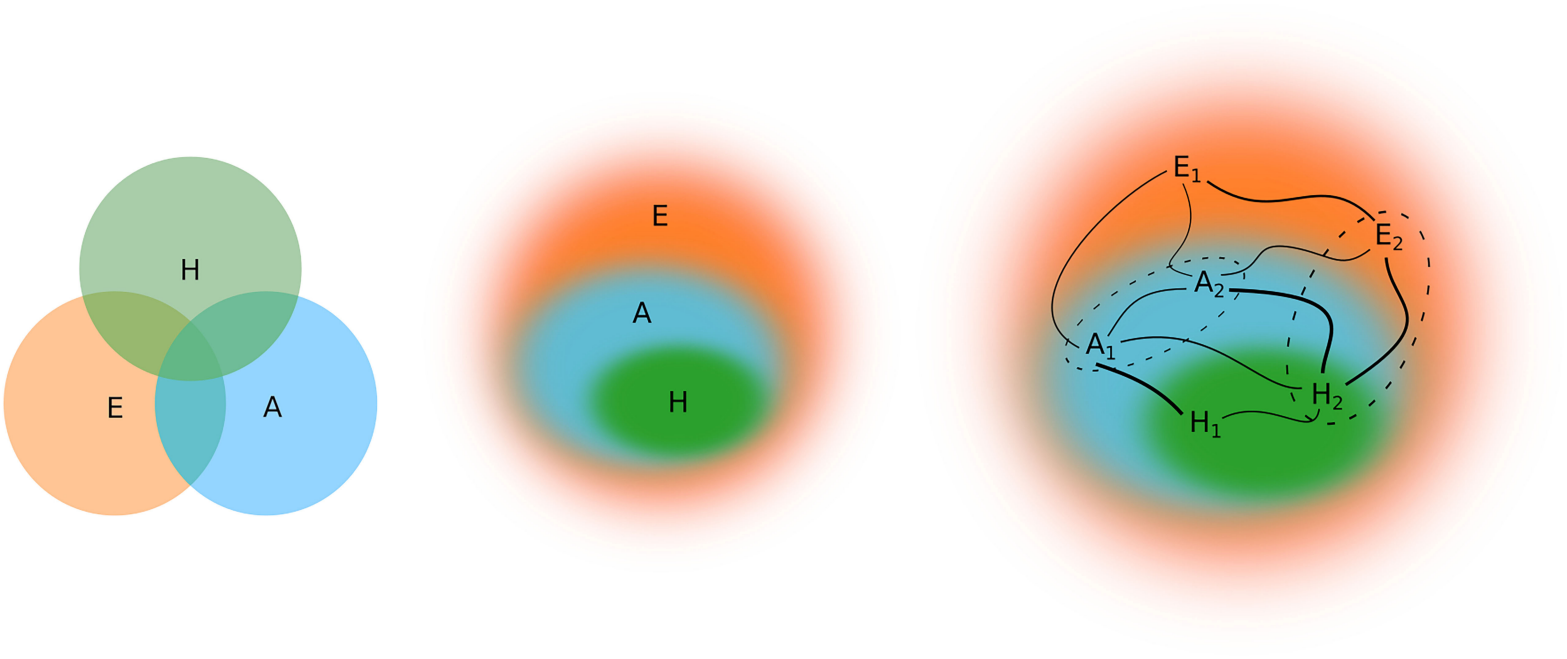
The conventional scheme partially intersect human, animal, and environmental health. In the proposed scheme of One Health, there is subsumption (*One* Health, inclusiveness, internal environments with blurred boundaries), differentiated relations (edges of different thickness), and plurality (indexed elements). a: non-human animal (species/collective/individual/intraindividual); h: human-animal (collective/individual/intraindividual); and e: vegetables, other living beings, inanimate agents (species/collective/individual/intraindividual). Dashed boundaries show that there are many configurations for multispecies collection.

One Health of Peripheries, does not dogmatically cut animal taxonomy to leave the human species on one side, and a wide variety of species on the other side (Figure 1). Instead, there are multispecies collectives whose species-specific constitution depends on the health phenomenon in question; the division of animal taxonomy into “human” and “animal” is understood as a tool that may have didactic and strategic values and serve as semantic abbreviation; however, the uncritical use of this tool conflates the division with a constant of “nature” and hides its biopolitical consequences.

In One Health of Peripheries, the environment is not understood as an external domain related or partially intersected by the human and animal domains. It is composed of multispecies collectives, so multispecies studies can help to think about it (10–12, 59); the environment is a set of relations and agents located by them; entanglements; agents that even as “individuals” reveal internal environments of microbiota; complex assemblages of holobionts (60). It is an environment without the dual ontology separating “human society” and “nature” (10, 12, 61, 62).

There are many holistic approaches to promote the health of such an environment. Many indigenous peoples have lived over centuries with a sense of integration reflected in sustainable and respectful environmental practices. Agroecology has learned from them, incorporates contemporary technologies, and brings equity to the center (63, 64). Living cities, recombinant ecosystems, and other movements of sustainable urban systems offer alternatives for cities (65–67). However, the colonial mentality and capitalist order deplete resources and marginalize collective endeavors driven by well-being instead of profit. Thus, breaking marginalizing apparatuses is as crucial here as elsewhere.

Besides the substantial difference between conventional One Health and One Health of Peripheries as conceptual frameworks, the last departs from the first in other directions. One Health of Peripheries is a polysemic expression with an ontological, an epistemological, and a practical sense. The first sense refers to attributes determining the well-being and suffering of peripheral multispecies collectives: a state, a process, the realization of capacities (note that capacity is a key notion in health promotion). The second problematizes the marginalizing apparatuses that determine health. The third encompasses practices against marginalization that informs and reinforces peripheral resistance and learns from it.

These three senses are not independent; each one is inherent to the others. While understanding and practice are attributes of multispecies collectives, attributes and understandings are practices and transformed by practice. Simultaneously, understanding gives sense to attributes and practices. This polysemy has material consequences, as theorizing new attributes lead to other practices to pursue the wellness of marginalized multispecies collectives.

The emphasis on marginalizing apparatuses has as a corollary the existence of an heterogeneous peripheral cartography. Thus, what follows in this section is an outline of peripheral regions that are anything but the whole cartography. Choosing some peripheries and not others is not an unproblematic decision; it can reinforce marginalization. Indeed, we cannot do everything simultaneously, but for that very reason, we must problematize what is at stake in prioritization. My decision is somehow arbitrary and shaped by my background. Nevertheless, I hope it sheds some light on pathways to identify and fracture even the margins I omit.

### Neglected Diseases

With renowned institutions listing neglected diseases, it is easier to see how the pharmaceutical industry disregards the needs of unprofitable populations. However, stressing diseases might divert attention from a fundamental point of neglect. Many tourists have information about the safer travel periods to avoid malaria, access to preventive medication, and health insurance to receive the best available treatment regime if they got infected. Rabies vaccine has been around for decades, but approximately 60 thousand humans die of rabies each year, mainly in the global South. Thus, not anybody with a neglected disease is neglected and what is at stake is not only the existence of pharmaceutical treatments.

The fundamental neglect resides on multispecies collectives and demands more than outreach policies. People representing those collectives need effective political inclusion; plural education to solve their problems and sustainably build their communities; food security and sovereignty; the multispecies collectives need more-than-human health systems and decolonial programs for caring ecosystems.

### Domestic Violence

Violence is a cause of morbidity and mortality, and among the approaches to address its complexity, it is the prevention of violence against animals. Conviviality with companion animals is growing, and in some countries, there are more dogs and cats than children in households (68, 69). In multispecies homes, animals enter into family dynamics and can be victims of domestic violence. The violence against them is related to the violence against children and women (70–74). In addition to victims, animals are instruments of coercion used by perpetrators to cause more suffering and control their human victims (75–79).

Domestic violence does not stem exclusively from individual psychological factors. Lifestyles, conditioned by processes of social reproduction, favor or protect against domestic violence, depending on their configuration. Therefore, it is important to consider the relationship between social vulnerability, interpersonal violence, and violence against companion animals (80–84).

The investigation of violence against animals helps to detect domestic violence cases involving several victims and broadens the understanding of the perpetrators' psychological profile (73). Furthermore, animals can promote collective care and self-care to counter violence (85, 86). However, the effective prevention of domestic violence must address social vulnerability and its social determination, in the broad sense, without being restricted to economic poverty and exploring underlying marginalizing apparatuses. It must dismantle the patriarchal apparatus underlying domestic violence. Domestic violence in One Health of Peripheries is socially determined, affects humans and animals, and has institutionalized dimensions.

### Geographic Peripheries

Geographic peripheries are heterogeneous, encompassing countries, areas circumscribed within countries, and cross-border regions such as rural areas, indigenous territories, and favelas. Taking the last as an example, we can see how geographic marginalization also circumscribes epidemiologic profiles. *Favela* is a term with pejorative connotations, unsolved by euphemisms. It refers more directly to the geographically delimited precariousness ensuing from the historical exploitation and concentration of wealth. Simultaneously, its polysemy point to the constant meaning-making and remaking from within; to the place from which resistance, creativity, and sensitivity produce other epistemologies and lifestyles.

The favelas challenge conventional census methods and thus receive differentiated treatment, starting from their identification. For instance, the Brazilian Institute of Geography and Statistics (IBGE) defines favelas as places with at least 51 housing units irregularly occupied, under urban irregularities, or lacking essential public services (87). It calls them subnormal agglomerates. Census definitions, although limited, give an idea of quantity. There were 6,329 favelas in which 6% of the Brazilian population lived in 2010. The State of São Paulo had the highest concentration of households in favelas (23.2%), including ~11% of its metropolitan population (87). Thus, health problems affecting favelas compromise millions of individuals in the country. Globally, projections suggest that in 2030 the human population will be 8.1 billion, 5 billion (61.7%) will live in urban areas, and 2 billion (24.7%) will live in favelas (88).

The neglect of favelas continues worldwide. The health in favelas is different from the urban health and health in poverty because not all people living in favelas are poor, and not all poor people in cities live in them (89). The favelas' contextual effects on health are mediated by imposed risks and the lack of resources (money, time, infrastructure, knowledge), establishing a vicious circle of vulnerability due to the increased burden of diseases that compromises the individuals' opportunities for economic and social inclusion.

The favelas' contextual effects impinge on multispecies collectives, and this is even more neglected. Animals are exposed and vulnerable to pollution, humidity, darkness, insufficient ventilation, malnutrition, and high population densities. There is a need to promote animal health for the sake of the animals but also for the sake of humans living with them. The life cycle of animals is shorter than in humans. Its monitoring contributes to the early detection of chronic diseases and other health problems resulting from exposure to unhealthy environments (90, 91). As favelas' boundaries are not hermetic and do not entirely restrict their contextual effects, improving their health reflects outside them. Favelas are peripheral but not isolated. Turning favelas into healthy places reduce infectious diseases, the need to use antibiotics, and thus antimicrobial resistance, one of the top ten global health problems according to the WHO. But as with any periphery, that turn requires structural changes, the dismantling of the underlying marginalizing apparatuses.

### Homelessness

“Homelessness” usually refers to the condition of humans without a permanent residence, a dynamic situation that can vary from 1 day to a lifetime, depending on the availability of social and economic resources to have access to such permanent residence.

Homelessness is a structural problem of social organization around private property, worsened by the precariousness of working conditions and welfare policies. However, it also results from other processes, such as the abandonment of homes to escape domestic violence or home dynamics incompatible with drug abuse, psychiatric illnesses, and other conditions.

In addition to humans, companion animals can turn homeless due to abandonment or because they got lost. They may be born homeless, remaining as such for the rest of their lives or until rescue.

Dogs and cats are still properties, and therefore their homelessness also represents a private property problem. On the one hand, the legal consequences of abandoning an animal property might not be sufficiently persuasive to avoid animal abandonment. On the other hand, the property status might reduce and even eliminate the moral responsibility regarding animal abandonment.

Although the processes that lead humans and companion animals to homelessness are different, some effects are similar regardless of the species. Homeless individuals suffer abuse. Adversities (climatic, nutritional, emotional) cause suffering and compromise the immune system, thus adding to the lack of hygiene that predisposes to infectious diseases, worsened by the lack of access to health services.

In their marginalized condition, homeless humans and dogs find each other and create emotional bonds (92, 93). Humans even prioritize dogs when sharing available food (94), and may prefer to remain on the streets than stay overnight in places that do not accept their canine companions (95). Citing Sakelaropoulos et al. (96), Taylor describes the humans' emotional bonds with cats and even rats (93). The latter and other synanthropic species live on public spaces and pose specific challenges that increase the health complexity of multispecies collectives living on the streets.

Direct actions on homeless multispecies collectives could involve networks of shelters and adoption programs for humans (mainly children in the case of adoption) and companion animals, as well as contraceptive and “humanitarian” elimination programs for synanthropic populations. These actions complement but do not replace structural approaches of health promotion and disease risk prevention.

Regardless of their species, the homeless are members of the living cities conceptualized in critical geography (65). One Health in the urban context turns out to be the health of these living cities, and their improvement demands special considerations about homelessness. First, promoting lifestyles as opposed to the conditions that lead humans and companion animals to homelessness. Second, urban planning to promote biodiversity; planning for the so-called recombinant ecosystems and green cities (65, 67).

### Agribusiness Externalities

Ending hunger is one of the United Nations' Sustainable Development Goals (97). Agribusiness has responded to such a goal by intensifying production, reducing food prices, generating jobs, and contributing to Gross Domestic Product (GDP). However, qualifying that response requires taking externalities into account. Although some externalities are gaining visibility, others remain peripheral.

The Intergovernmental Panel on Climate Change (IPCC) concluded, with a high level of confidence, that “climate change is expected to lead to increases in ill-health in many regions and especially in developing countries with low income, as compared to a baseline without climate change” (98). Greenhouse gases (GHG) are the leading cause of climate change (99), and farm animals are the largest source in agriculture (100). Furthermore, single-crop farming is another source of GHG itself. Its expansion often leads to more emissions due to the intensification of farm animal production to compensate for the loss of pastures (101).

The expansion of agricultural frontiers reduces biodiversity and increases the risk of many zoonoses occurrence (102). However, zoonoses control proposals are typically biomedical or focused on proximate risk factors. They hardly question the food production systems' *status quo*, thus losing the opportunity to find more favorable scenarios in terms of zoonoses, protection of biodiversity, and other externalities. Moreover, the loss of biodiversity is rarely understood as a direct One Health problem, characterized by increases in mortality rates of multiple animal and plant species (103), *and* losses of multiple ethnic collectives.

Water consumption and pollution are other externalities of agribusiness. In Brazil, for example, it is estimated that land irrigation consumes 72% of the country's water supply (104), and feeding farm animals consume 79% of the cultivated protein (105). Simultaneously, the water network did not serve 33.2 million people in 2018 (106). In animal production systems, sources of water pollution include pharmaceutical residues (including antibiotics), heavy metals, chemicals, excrement, and pathogens; as for crops, in addition to heavy metals and chemicals, pesticides with carcinogenic potential are of particular concern (107).

Agribusiness creates jobs and contributes to GDP. However, it matters what kind of jobs, in a context of employees with little bargaining power against growing oligopolies (108, 109). For instance, in subaltern countries, subsidies persuade smallholders to submit themselves to exploitation by transnational corporations at the expense of agrarian reforms to promote diversified agriculture equitably (108, 109). Meanwhile, in rich countries, unhealthy conditions in intensive production systems difficult the recruit domestic workers, which has been circumvented by hiring immigrants, including those who are not authorized to work. (110, 111).

Unhealthy work can occur for several reasons. In the production of fruits and vegetables, pesticides are potential carcinogens (107, 112, 113). In intensive animal production systems, toxic gases, vapors, and particles pollute the air and cause respiratory diseases (114–116). Farm environments and slaughterhouses can predispose to physical trauma, depression, and drug use (50, 111). Stressful and overpopulated environments also predispose to animal diseases, and their treatment with antibiotics results in antimicrobial resistance affecting human workers and their families (117–120). In slaughterhouses, the mass killing of animals is a violent job that can affect the employees' mental health, and slaughterhouse employment has been causally linked to increased crime rates in communities neighboring such slaughterhouses (121).

The externalities on farm animal wellness have been explored elsewhere (109, 122). Here I want to emphasize that despite recent theoretical advances on multispecies justice and labor issues involving animals (13, 15), forcing animals to produce continues without considering labor rights for them. Farm animals are subjected to a commodification strategy that transforms the violence perpetrated on sentient beings into procedures to increase production efficiency.

While happy farm animals appear in bucolic images (in children's books and milk packages) and Ag-gag laws prevent the investigation and disclosure of animal abuse (123, 124), the real farm animals are pushed to their physiological limit, constantly expanded by genetic, medical, and pharmacological technologies. Billions of these animals are slaughtered, requiring hasty procedures that challenge labor safety and animal suffering mitigation. Moreover, cruelty procedures continue in use: male chicks shredded alive when the objective is egg production; sows housed in cells that prevent them from turning their bodies; small cages that do not allow birds to extend their wings; prematurely broken mother-offspring bonds; routine amputation and without anesthesia of beaks, teeth, horns, and tails to increase confinement density and avoid cannibalism ensuing from the stressing environment.

Agribusiness produces externalities protected by strategies of governmentality (109). It destroys the environment and uses cruel methods against animals. Simply talking about job creation and GDP contribution does not say anything about the working conditions or the profit distribution. Externalities, including subsidies, outweigh the final prices paid by consumers of agribusiness' commodities and threaten global sustainability. Agribusiness marginalize multispecies collectives inside and outside farms.

## Social Determination of Health

There are discussions about health complexity beyond biomedical issues. In Latin America, social medicine (nowadays collective health and critical epidemiology) has developed conceptual frameworks for the social determination of health since the 1970s. After the turn of the century, the WHO has popularized a conceptual framework of the social determinants of health. Despite criticisms from critical epidemiology to the WHO proposal for being in practice more complicit with the *status quo* structuring inequities (1, 125, 126), both positions point to the need to transcend biologism and individualism in health, but they also reduce the social to the human domain. However, some approaches to One Health show that reducing social relations to humans is misleading (23, 52, 127), whereas biopolitics and sociology set background to think a more-than-human social determination of health (9–11, 30, 128–130).

In the WHO's conceptual framework, structural determinants create health inequities through intermediary determinants (3). The structural determinants refer to the mechanisms by which political, economic, and social contexts generate “hierarchies of power, prestige, and access to resources” (3). The intermediary determinants are material and psychosocial circumstances, behavioral and biological factors, and the health system itself; they are a consequence of individuals' hierarchical positions. They are also the cause of exposures and vulnerabilities leading to health inequities (3).

Social cohesion and social capital are considered as both structural and intermediary determinants while the health state affects individuals' opportunities and thus feedback into the hierarchical structure (3). In short, it is a conceptual framework of causal nature where structural determinants have a position of precedence and prominence. The identification and measurement of the hypothetical effect of causal factors inform decision-making to reduce health inequities.

The social determination of health theorized in Latin America is not synthesized in a single reference. However, a common feature of different perspectives is that social determination is a category of critical analysis (1, 2, 131, 132). According to Samaja, social determination is a historical and ongoing process through which social hierarchy levels are “self-produced and reproduced, generating tensions and conflicts that motivate actions of restoration and transformation” (132) [translation is mine]. A given hierarchical level *reproduces* itself as a whole, regulating its parts (levels subsumed by it) to maintain the whole structure (132). However, the regulation is not absolute, and the relative autonomy of the parts is a source of change that *produces* new wholes (levels subsuming them) (132).

In this dialectic movement between regulation and relative autonomy, healthy and unhealthy forces configure epidemiologic profiles characteristic of the different hierarchical levels and positions within the levels (131). For instance, the family is one of such levels. The relative autonomous lifestyles of family members, as well as the regulations from higher social organization levels (community, political-administrative territorial divisions, contractual associations, and other institutions), determine their epidemiologic profile.

Despite fundamental differences between the two conceptual frameworks, they intersect at two points. Both identify a structural dimension (socioeconomic and political context in the social determinants; social production and reproduction in the social determination) and the ensuing hierarchy that imposes constraints on individuals according to their hierarchical position. Both point to the triple inequity of health determined by class, gender, and race/ethnicity.

One Health of Peripheries also intersects these points. The first from a biopolitical perspective in which the political is neither an external precursor of hierarchies nor an instrument monopolized by the most privileged hierarchical levels. The political is the relationships among individuals, the hierarchical order itself, it is realized and not owned, it is the foucauldian micro-physics of power (133) involving animals. Therefore, One Health of Peripheries participates in the second intersection in its theorizing of multispecies forms of health inequity.

Structural One Health is another helpful reference that goes beyond proximate causes to explore the crucial role of agribusiness in the production of zoonoses and pandemics through circuits of capital (52). However, it is worth noting that structural One Health and One Health of Peripheries differ. First, there is a difference of scope because One Health of Peripheries extends beyond infectious diseases. Second, structural One Health stresses more extensive empirical causal processes, whereas One Health of Peripheries agree with the need for more comprehensive causal explanations but stresses dialectical process to overcome the limitation of causal reasoning and empirical evidence. Third, power relations and health inequities are explicit multispecies phenomena in One Health of Peripheries. It is beyond the scope of this paper to explore the details of the (eco)social determination of One Health of Peripheries, so I will leave that for future works.

## Field of Praxis

Field and habitus are bourdieusian concepts incorporated in collective health. From them, we can think about health practices and knowledge as elaborated by subjects conditioned by symbolic structures like language and culture that allow and shape their representations and actions. Therefore, health is for health practitioners what they can know about it, so transforming the conditions that make knowledge possible changes health. In other words, the transformation of symbolic structures is also a health practice and affects health.

Practices are produced, perceived, and appreciated by *habitus*, a system of schemes “constituted in the course of collective history and acquired [and transformed] in the course of individual history” (8, 134) [translation is mine]. Individuals' *habitus* depends on hierarchies, so individual's perceptions, knowledge, and practices reveal their position and shape their relationships with individuals in other hierarchical places.

The field is the social space constituted by hierarchical relationships that condition the *habitus* and gain from this its meaning and value (135). In the filed, cooperation and conflict preserve or transform hierarchies. The most privileged positions have more capital—economic, cultural, social, and symbolic—to shape and legitimize hierarchies according to their interests. These interests are not necessarily conscious because, as part of the *habitus*, they are inculcated in “institutionalized spaces (family, school) by specialized agents who impose arbitrary norms using disciplinary techniques” (8) [translation is mine].

Peripheral positions “intervene as a passive, contrasting reference point” (8) [translation is mine]. Here is again the contrasting position of animals; those who want more capital to fight and legitimize their interests need a “social promotion experienced as an ontological transformation or as a process of civilization, a leap from nature to culture, from animality to humanity” (8) [translation is mine]. Thus, our relationships with animals are among the conditions of possibility of the habitus we acquire, and this, in turn, gives meaning and value to multispecies assemblages.

Depending on the *habitus* and the field, one will see, among others, unfitted mads who deserve their misfortunes, or psychiatric patients who can become more productive when receiving treatments provided by the pharmaceutical industry, or unhealthy exploitation regimes by way of progress. One will see pests and reservoirs of infectious agents that threaten public health, or multispecies collectives that share susceptibilities, in need of comprehensive health policies. Therefore, what enters into the health field and the way it enters is a social process.

Health practice is not neutral and can reinforce inequities. On the contrary, promoting One Health of Peripheries is an explicit commitment to reduce more-than-human inequities. Thus, the field of practice for such promotion is more specific; it is a field of praxis. Here I take praxis from Paulo Freire as reflexive action against oppression, toward liberation (136). Praxis as action informed by knowledge about the pathological effects of marginalization, and knowledge built on actions against marginalization.

In the field of collective health, there is extensive reference to “health promotion” and “life preservation” (137), non-anthropocentric perspectives (1), and “diversity of objects and theoretical discourses, without recognizing any hierarchical and evaluative perspective about them” (138) [translation are mine]. However, any generic reference to life or health is systematically pointed to the human, overlooking that life and health are more-than-human. This is a blind spot of collective health, brought to light by the praxis of One Health of Peripheries.

As a subfield of health, collective health does not need to cover everything that concerns health, and in this sense, it could be limited to the human. However, if collective health is transdisciplinary (139), concerned with the social determination of health (1) and aims at the “production of an expanded knowledge of health” (140) [translation is mine] it should promote One Health of Peripheries.

## Conclusion

One Health of Peripheries is experience, understanding, and transformation to improve the wellness of marginalized multispecies collectives. One Health of Peripheries is about breaking margins to pursue multispecies justice.

Biopolitics and other critical perspectives offer conceptual tools to understand why marginalizing apparatuses determine most of the burden of ill-health and why we need multispecies intersectionality to achieve equitable alternatives.

Biological solutions stripped from the more-than-human social reality will not solve the remarkable challenges posed by mainstream One Health. Indeed, insisting on supposed apolitical and non-ideological epidemiologic settings of transmissible and physiopathological processes is part of the problem, just as pretending that all we need is a strong pharmaceutical industry supported by patents, intersectoral collaborations between “symmetrical” parties, and good deeds of the global North toward the global South.

The social determination of health is a comprehensive framework to embrace health complexity. However, it has a blind spot: One Health of Peripheries. The anthropocentrism of collective health perpetuates marginalization and limits the reach of health promotion.

One Health of Peripheries takes advantage of more-than-human biopolitics, One Health, collective health, and other sources of knowledge to inform the commitment of taking multispecies collectives out of peripheries. Such diversity inevitably incorporates theoretical difficulties.

It is worth noting that I am talking about One Health *of* Peripheries instead of One Health *on* Peripheries. That makes the commitment stronger as it is not purported to be a top-down endeavor. As a side comment, it was working *with* communities in favelas that I felt the need for a different theoretical background. Thus, I ended up trying to give sense to One Health of Peripheries.

The plurality of (academic, popular, and traditional) knowledge and the decolonial commitment of One Health of Peripheries need an explicit agenda. In another paper, we frame colonial apparatuses of marginalization, elaborate on how the epistemologies of the South are suitable to work with plural knowledge, and propose seven actions to promote One Health of Peripheries (18).

## Data Availability Statement

The original contributions presented in the study are included in the article/supplementary material, further inquiries can be directed to the corresponding author/s.

## Author Contributions

The author confirms being the sole contributor of this work and has approved it for publication.

## Conflict of Interest

The author declares that the research was conducted in the absence of any commercial or financial relationships that could be construed as a potential conflict of interest.
